# Novel Implications of DNA Damage Response in Drug Resistance of Malignant Cancers Obtained from the Functional Interaction between p53 Family and RUNX2

**DOI:** 10.3390/biom5042854

**Published:** 2015-10-23

**Authors:** Toshinori Ozaki, Mizuyo Nakamura, Osamu Shimozato

**Affiliations:** Laboratory of DNA Damage Signaling, Chiba Cancer Center Research Institute, Chiba 260-8717, Japan; E-Mails: mnakamura@chiba-cc.jp (M.N.); oshimoza@chiba-cc.jp (O.S.)

**Keywords:** AKT, anti-cancer drug, cancer stem cell, CD133, cell cycle arrest, cell death, DNA damage, drug resistance, p53 family, RUNX family

## Abstract

During the lifespan of cells, their genomic DNA is continuously exposed to the endogenous and exogenous DNA insults. Thus, the appropriate cellular response to DNA damage plays a pivotal role in maintaining genomic integrity and also acts as a molecular barrier towards DNA legion-mediated carcinogenesis. The tumor suppressor p53 participates in an integral part of proper regulation of DNA damage response (DDR). *p53* is frequently mutated in a variety of human cancers. Since mutant p53 displays a dominant-negative behavior against wild-type p53, cancers expressing mutant p53 sometimes acquire drug-resistant phenotype, suggesting that mutant p53 prohibits the p53-dependent cell death pathway following DNA damage, and thereby contributing to the acquisition and/or maintenance of drug resistance of malignant cancers. Intriguingly, we have recently found that silencing of pro-oncogenic *RUNX2* enhances drug sensitivity of aggressive cancer cells regardless of *p53* status. Meanwhile, cancer stem cells (CSCs) have stem cell properties such as drug resistance. Therefore, the precise understanding of the biology of CSCs is quite important to overcome their drug resistance. In this review, we focus on molecular mechanisms behind DDR as well as the serious drug resistance of malignant cancers and discuss some attractive approaches to improving the outcomes of patients bearing drug-resistant cancers.

## 1. Introduction

Cells are continuously exposed to endogenous as well as exogenous DNA damage-inducing stimuli including oxidative stress, ultraviolet (UV), ionizing radiation (IR), oncogene activation and/or anti-cancer drugs. The appropriate DNA damage response (DDR), which is a coordinated series of cellular events, contributes to the maintenance of genomic stability and acts as a molecular barrier against carcinogenesis. Thus, the defects in DDR result in genomic instability and then promote the development of cancer. Upon DNA damage, one of the initial cellular events is an auto-phosphorylation (activation) of ATM (ataxia telangiectasia mutated). A phosphorylated form of ATM at Ser-1981 (referred to as p-ATM) then phosphorylates histone variant H2AX at Ser-139 (referred to as γH2AX) to mark the sites of DNA damage (nuclear foci) [1]. The appearance of γH2AX has been widely used as a specific DNA double-strand break (DSB) marker [2]. After p-ATM-mediated phosphorylation of H2AX following DNA damage arising from radiation and/or anti-cancer drugs, MDC1 (mediator of DNA damage checkpoint protein 1)/NFBD1 (nuclear factor with BRCT domains protein 1) (henceforth NFBD1) binds to γH2AX and serves as an anchor protein for the subsequent recruitment of MRN (Mre11-Rad50-Nbs1) DNA damage sensor complex [3,4,5].

Meanwhile, MRN complex is a dynamic macromolecular machinery, which acts at the initial step of DNA double-strand break repair, and has an impact on homologous recombination repair as well as non-homologous end-joining. Among the components of MRN complex, Rad50 is the largest protein with ATPase activity [6]. Mre11 has a single-strand DNA endonuclease and 3'–5' double-strand exonuclease activities [7]. Its C-terminal domain is responsible for protein–protein and protein–DNA interactions. ATP-dependent conformational change in Rad50 ATPase domain regulates Mre11 nuclease activity [8]. Meanwhile, Nbs1 contains a fork head-associated (FHA) domain and two BRCA1. C-terminus (BRCT) domains, which are required for the complex formation with phosphorylated proteins such as ATM, NFBD1 and Mre11 [9]. It is worth noting that MRN complex further stimulates ATM activity, which results in a rapid spreading of γH2AX around the sites of DNA damage, and thereby amplifying DDR signal [10,11,12]. During the early phase of DDR, cell cycle arrest takes place to facilitate DNA repair. When cells are exposed to serious DNA damage, cells undergo cell death. If DNA lesions are not repaired or repaired incorrectly, the genomic instability is induced and causes carcinogenesis.

The representative tumor suppressor p53 is a nuclear sequence-specific transcription factor [13,14,15]. In response to DNA damage, p53 rapidly accumulates and is activated in cell nucleus through the sequential post-translational modifications such as phosphorylation (Ser-15, Ser-20 and Ser-46) and acetylation (Lys-373 and Lys-382). Activated form of p53 transactivates a number of its target genes implicated in the induction of cell cycle arrest (*p21^WAF1^* and *14-3-3*σ), DNA repair (*GADD45*), cellular senescence (*p21^WAF1^*) and cell death (*BAX*, *NOXA* and *PUMA*), and thereby exerting its growth-suppressive and pro-apoptotic functions to finally eliminate cells with seriously damaged DNA. Hence, the sequence-specific transactivation ability of p53 is tightly linked to its pro-arrest and pro-apoptotic functions [13,16]. Intriguingly, *p53*-deficient mice developed spontaneous tumors [17]. Moreover, extensive mutation searches demonstrated that *p53* is frequently mutated in over 50% of various human cancers [18]. Among *p53* mutations, 90% of its mutations are detectable within the genomic region encoding its sequence-specific DNA-binding domain. As expected, mutant forms of p53 with a longer half-life lack the sequence-specific transactivation ability and participate in the acquisition of the pro-oncogenic potential. Indeed, cancer cells expressing mutant p53, which has a dominant-negative effect on wild-type p53 and/or a pro-oncogenic property (gain-of-function), exhibit drug-resistant phenotype [19,20,21]. Together, p53 stands at the crossroad between cell survival and death in response to DNA damage.

RUNX2 is one of runt-related sequence-specific transcription factors (RUNX family), and has been considered to be a master regulator for osteoblast differentiation as well as bone formation [22,23]. In addition to its role in the regulation of osteogenesis, it has been shown that the expression level of *RUNX2* is higher in a variety of human cancer tissues including pancreatic, breast, colon, prostate cancers and osteosarcoma as compared with that in their corresponding normal ones, indicating that RUNX2 has an oncogenic potential [24,25,26]. Consistent with this notion, RUNX2 has an ability to transactivate invasion- and/or metastasis-related genes such as *Survivin*, *MMP-2*, *MMP-9* and *VEGF* [27,28,29,30,31,32,33,34]. Recently, we have found for the first time that depletion of *RUNX2* in osteosarcoma-derived cells significantly enhances their adriamycin (ADR) sensitivity [35]. Based on our results, RUNX2 attenuated p53-dependent cell death pathway in response to ADR. Together, it is likely that RUNX2 is tightly linked to drug-resistant phenotype of malignant cancers.

The cancer stem cell (CSC) hypothesis has become increasingly accepted to provide a clue to understanding the precise molecular mechanism(s) behind cancer initiation, progression, metastasis and recurrence [36,37,38]. According to this model, a small sub-population of the heterogeneous cancer cells has a greater potential to initiate distant metastasis and acquire drug resistance. Recent extensive studies demonstrated that CSC-like cells are found in brain, breast, colon, lung, liver, pancreas, ovarian, head and neck, melanoma and prostate cancers [39]. Isolation of CSCs is dependent on their specific molecular markers. A growing body of evidence suggests that there exist several molecular markers for CSCs such as CD44, CD24, ESA, CD13 (aminopeptidase N), CD133 (also known as prominin I) and ALDH1 (aldehyde dehydrogenase 1) [40,41,42,43,44]. Among them, the earliest identified marker is CD133 [45]. Although the functional significance of CD133 in CSCs’ biology remains unclear, it has been described that CD133-positive glioblastoma cells are resistant to anti-cancer drugs such as temozolomide, carboplatin, VP16 and taxol [46]. Of note, CSC-enriched fractions prepared from prostate cancer tissues highly expressed *RUNX2* as well as its target gene *Survivin*, implying that RUNX2 might be associated with the biology of CSC in prostate cancer [47,48].

**Figure 1:**
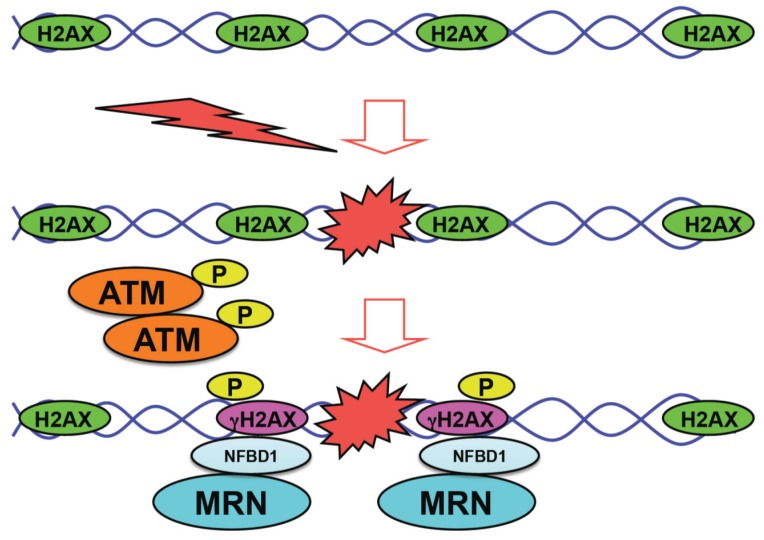
Initial molecular events in response to DNA damage. Upon DNA damage, activated form of ATM (p-ATM) phosphorylates histone H2AX (γH2AX) to mark the sites of DNA damage (nuclear foci). After nuclear foci formation, NFBD1 binds to γH2AX and then MRN complex is efficiently recruited onto the sites of DNA damage.

In brief, the initial molecular event during DDR is the auto-phosphorylation of ATM, which phosphorylates histone H2AX at the sites of DNA damage. Subsequently, NFBD1 binds to γH2AX followed by the recruitment of macromolecular DNA repair machinery including MRN complex, and thereby damaged DNA is repaired. Then, cells with accurately repaired DNA re-enter the normal cell cycle (Figure 1).

Here, we give an overview of the mechanistic basis underlying the development of serious drug-resistant properties of malignant cancers, and also discuss novel, promising strategies for their treatment.

## 2. The Representative Tumor Suppressor p53

The question is how cell fate determination (cell survival or death) could be regulated in response to DNA damage. One of the key proteins, which stands at the crossroad between cell survival and death, is the tumor suppressor p53.

p53 is a nuclear sequence-specific transcription factor containing an N-terminal transactivation domain (TA), central sequence-specific DNA-binding domain (DB) and C-terminal oligomerization domain (OD) [13,14,15]. As expected, p53 transactivates various target genes implicated in the induction of cell cycle arrest, DNA repair, cellular senescence and/or cell death. Since p53 is a dangerous protein with a pro-apoptotic potential, p53 is inactivated and also kept at an extremely low level under normal physiological conditions (quite short half-life, 20 min). Upon DNA damage, p53 is quickly stabilized and activated in cell nucleus through the sequential post-translational modifications such as phosphorylation and acetylation. Accumulating evidence strongly indicates that the expression of p53 is largely regulated at protein level but not at mRNA level [49,50,51].

E3 ubiquitin ligase MDM2 binds to N-terminal TA domain of p53 and efficiently ubiquitinates its C-terminal Lys residues. Poly-ubiquitinated p53 is then subjected to proteasome-dependent proteolytic degradation. In addition to MDM2, Pirh2, COP1 and ARF-BP1 have been shown to target p53 by ubiquitination for degradation through proteasome [52,53,54]. MDM2 simultaneously masks TA domain of p53 and inhibits its sequence-specific transactivation ability [55,56]. *MDM2* is in turn transcriptionally induced by p53, and forms an auto-regulatory feedback loop, which regulates p53 expression level [57,58]. DNA damage-induced phosphorylation of p53 at Ser-15 mediated by p-ATM promotes the dissociation of MDM2 from p53, and thereby dramatically increases its half-life [59,60]. Besides p-ATM-mediated p53 phosphorylation at Ser-15, histone acetyltransferase p300/CBP, which also acts as a transcriptional co-activator, is associated with TA domain of p53 and then acetylates its C-terminal Lys residues to enhance its transactivation ability following DNA damage [61].

As mentioned above, p53 induces cell cycle arrest and/or cell death in response to DNA damage. It is well known that p53-dependent cell cycle arrest requires transactivation of *p21^WAF1^* (G1/S) and/or *14-3-3*σ (G2/M). Whereas, p53-dependent cell death is mediated by mitochondrial dysfunction through the up-regulation of *BAX*, *PUMA*, *NOXA* and/or *p53AIP1*. Unfortunately, the molecular basis, which could determine the initiation of either p53-mediated cell cycle arrest or cell death program, remains unclear. The earlier studies indicate that cell cycle-related gene promoters are activated by p53 under low levels of DNA damage, whereas p53-target cell death-related gene promoters require a higher level of DNA damage [62]. Oda *et al.* found that, upon repairable DNA damage, p53 is phosphorylated at Ser-15 as well as Ser-20, and then recruited onto the promoter regions of cell cycle arrest genes such as *p21^WAF1^* [63]. When DNA damage is severe and repair is impossible, p53 is phosphorylated at Ser-46 and the resultant conformational change makes p53 to have a higher affinity to promoters of cell death-related genes such as *p53AIP1*. Therefore, a clear understanding of how p53 could select the pathways of cell cycle arrest or cell death following DNA damage provides a clue to developing a strategy to enhance drug sensitivity of malignant cancers.

One possibility is that p53-target promoters involved in cell cycle arrest have a higher affinity to p53, whereas the lower affinity target promoters are associated with cell death [63,64]. Another hypothesis is that the core promoter composition of p53-target genes plays a vital role in target gene selectivity [65]. Espinosa *et al.* have found that the amounts of RNA polymerase II complex bound to cell cycle-related gene promoters are larger than those of cell death-related ones [66]. Scala *et al.* reported that a highly stabilized p53 induces cell death, whereas a lower level of p53 triggers cell cycle arrest but not cell death in response to DNA damage, indicating that the amounts of activated p53 might determine cell fate [67]. As described above [63], phosphorylation of p53 at Ser-46 has an important role in the regulation of cell death. Consistent with these observations, it has been shown that HIPK2-mediated p53 phosphorylation at Ser-46 in response to UV exposure triggers transcriptional induction of pro-apoptotic p53-target genes [68]. In addition to phosphorylation, acetylation of p53 affects its target gene selection. For example, p53 acetylation at Lys-120 augments p53-dependent cell death [69,70,71], whereas its acetylation at Lys-320 promotes pro-arrest gene transcription such as *p21^WAF1^* but not pro-apoptotic genes [70,71].

Collectively, cell fate determination in response to DNA damage (cell survival or death) might be at least in part dependent on p53-target gene selectivity.

## 3. Mutant Forms of p53 and Anti-Cancer Drug Resistance

As reported previously [13], the sequence-specific transactivation ability of p53 is tightly linked to its DNA damage-mediated biological processes such as the induction of cell cycle arrest and/or cell death. Extensive mutation searches demonstrated that *p53* is frequently mutated in a variety of human cancers [72]. According to their observations, over 90% of *p53* mutations are detectable within the genomic region encoding its sequence-specific DNA-binding domain. Among them, there are six hot-spot mutations including Arg-175, Gly-245, Arg-248, Arg-249, Arg-273 and Arg-282. Mutant forms of p53 lack the sequence-specific transactivation ability [73]. Subsequent studies revealed that p53 mutants display a dominant-negative behavior towards wild-type p53 through the formation of hetero-oligomers with wild-type p53 and contribute to the acquisition of pro-oncogenic potential [15]. Transgenic mice expressing mutant p53 exhibited accelerated tumor development [74], and certain cancerous cells bearing *p53* mutations sometimes showed drug-resistant phenotype [19,20,21]. In addition, the expression level of mutant p53 in cancers has been shown to link to poor prognosis of the patients [75]. In a sharp contrast to a short-lived wild-type p53, mutant p53 has a prolonged half-life (2–12 h) due to the avoidance of MDM2-mediated proteasomal degradation [76], however, its precise molecular basis has not been fully understood. Hence, it is conceivable that the intracellular balance between the expression levels of wild-type p53 and mutant p53 might determine the cell fate in response to DNA damage.

**Figure 2:**
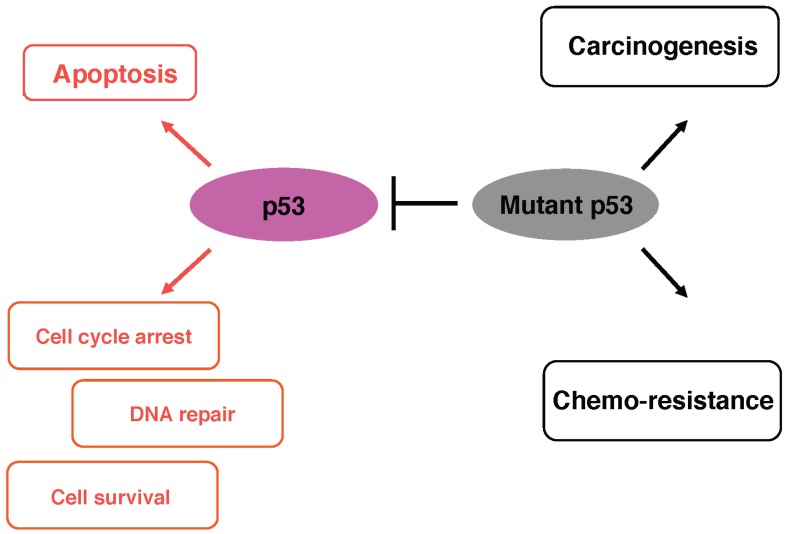
Functional interplay between wild-type p53 and mutant p53. Mutant p53 acts as a dominant-negative inhibitor against wild-type p53 and then contributes to carcinogenesis and drug-resistance.

Although mutant p53 lacks the sequence-specific transactivation ability, it has been described that p53 mutant has its own target genes distinct from those of wild-type p53 [77]. For example, mutant p53 regulates a number of genes implicated in carcinogenesis including *Myc*, *Fos*, *PCNA*, *IGF1R*, *EGR1*, *NFKB2*, *BCL-xL*, *IGF2* and *VEGFA* [78]. Recently, Kolukula *et al.* found that mutant p53 transactivates pro-oncogenic *SLC25A1* [79]. Unfortunately, no defined mutant p53-responsive element has been characterized so far. Further studies are required to adequately address this issue (Figure 2).

## 4. p53 Family Members p73 and p63

**Figure 3:**
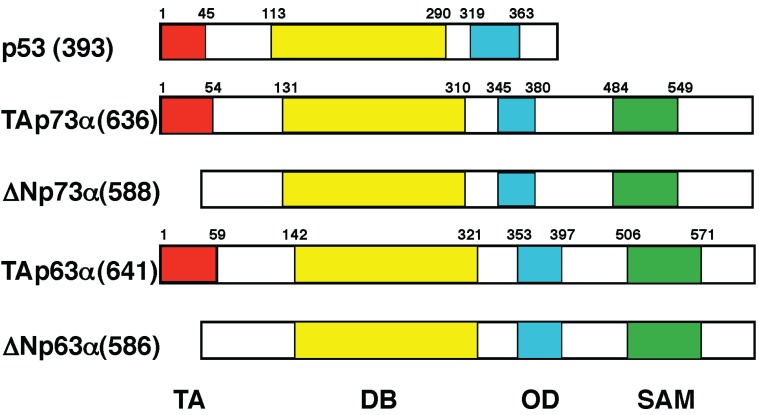
Structural comparison among p53 family members. Transcriptionally active p53, TAp73 and TAp63 contain the intact *N*-terminal transactivation domain (TA), central sequence-specific DNA-binding domain (DB) and *C*-terminal oligomerization domain (OD). In addition to these functional domains, TAp73 and TAp63 have the sterile α motif domain (SAM) implicated in protein–protein interaction. ΔNp73 and ΔNp63 lack N-terminal TA domain.

For a long time, *p53* has been believed to be a solitary gene. This classical point of view has been challenged by a discovery of novel human p53 homologues termed p73 and p63 [80,81,82]. At present, p53 family is composed of p53, p73 and p63. As deduced from their structural similarity to p53, p73 and p63 are involved in tumor suppression, which regulate cell proliferation, differentiation and death [83]. Like p53, p73 and p63 are induced in response to DNA damage, and transactivate an overlapping set of p53-target genes involved in the induction of cell cycle arrest and/or cell death [84,85]. In contrast to *p53*, *p73* and *p63* are rarely mutated in human cancers, suggesting that p73 and p63 are not the classical tumor suppressors [86]. Consistent with these observations, initial studies demonstrated that *p73*- or *p63*-deficient mice do not develop spontaneous cancers [87,88,89]. Notably, *p73* and *p63* encode multiple variants (TAp73, ΔNp73, TAp63 and ΔNp63) arising from alternative splicing and alternative promoter usage [80,81,90]. TAp73 and TAp63 are transcriptionally active valiants containing the intact *N*-terminal TA domains, whereas ΔNp73 and ΔNp63 are *N*-terminally truncated ones lacking the sequence-specific transactivation ability (Figure 3).

Like mutant p53, ΔNp73 and ΔNp63 display dominant-negative behavior towards TAp73 and TAp63 through hetero-oligomer formation, respectively, and are implicated in the acquisition of their oncogenic potential [82]. Consistently, forced expression of ΔNp73 promotes malignant transformation [91,92]. In particular, ΔN isoforms of p73 and p63 are aberrantly expressed in a variety of human cancers and their overexpression is associated with poor prognosis of the patients [93]. We have found for the first time that Δ*Np73* transcription is positively regulated by TAp73 through p53/TAp73-responsive element within Δ*Np73* promoter region [94]. Similar results were also reported by the other laboratories [95,96]. Therefore, it is likely that there exists a negative feedback regulation of TAp73 by its transcriptional target gene product ΔNp73 to avoid an inappropriate cell death by over-active TAp73 in response to DNA damage.

As described [87,88,89], the initial knockout studies demonstrated that *p73*- or *p63*-deficient mice do not develop spontaneous cancers, which might be attributed to the simultaneous disruption of both pro-apoptotic TA and pro-oncogenic ΔN isoforms. With this in mind, subsequent studies were performed using a mouse model in which the selective knockout of *TAp73* isoform but not Δ*Np73* isoform was designed [97]. According to their results, *TAp73*-deficient mice exhibited an increased cancer susceptibility and infertility, implying that TAp73 has a tumor suppressive role. Similarly, TAp63 has a critical role in preventing invasiveness and metastasis of epithelial cancers [98]. It is worth noting that p53-dependent cell death in response to DNA damage requires TAp73 and TAp63, whereas TAp73 and TAp63 are capable of triggering DNA damage-mediated cell death in the absence of functional p53 [99]. In fact, forced expression of TAp73 in *p53*-deficient human pancreatic cancer AsPC-1 cells resulted in massive cell death [100].

Upon DNA damage, TAp73 as well as TAp63 accumulates and is activated in cell nucleus. As described previously [101,102,103], TAp73 is phosphorylated by non-receptor tyrosine kinase c-Abl at Tyr-99 and its stability is significantly enhanced after DNA damage. Unlike p53, MDM2 prohibits TAp73-mediated transcriptional activation but does not affect its protein stability [104,105]. TAp73 and TAp63 are degraded through ubiquitin/proteasome mediated by E3 ubiquitin ligase Itch [106,107]. It remains elusive whether MDM2 could inhibit the transcriptional and pro-apoptotic activities of TAp63 [108,109]. In addition to the regulation of TAp73 at protein level, *TAp73* is transcriptionally regulated by E2F-1 [110,111,112,113]. Blattner *et al.* put forward that E2F-1 is up-regulated and promotes cell death after DNA damage [114]. On the other hand, the transcription of *TAp73* is repressed by the zinc finger/homeodomain-containing transcriptional repressor ZEB through ZEB-binding sites within the intron 1 of *TAp73* [115]. Together, E2F-1-induced cell death might be mediated at least in part by TAp73.

## 5. RUNX Family

As mentioned below, mammalian runt-related sequence-specific transcription factor (RUNX) family members including RUNX1, RUNX2 and RUNX3 are closely involved in carcinogenesis, and it is possible that there could exist a functional interaction between RUNX family and p53 family. Each of RUNX family members has a highly conserved sequence-specific DNA-binding domain (known as a Runt domain) and a distinct C-terminus, which contains both inhibitory and activation domains [116]. The Runt domain is associated with core-binding factor subunit-β (CBF-β), which is required for the tight interaction of RUNX proteins with their target sequences (5'-PuACCPuCA-3') (Pu indicates purines) [117]. Accumulating evidence strongly suggests that each of RUNX family members is involved in a distinct biological process. For example, *RUNX1* has been identified as part of the t(8; 21) chromosome translocation in acute myeloid leukemia (AML) and is responsible for the establishment of the hematopoietic stem cells [118,119,120]. *RUNX1* is the most frequent target for chromosomal translocation in AML, and *RUNX1* point mutations are found in hematological diseases such as AML as well as acute lymphocytic leukemia (ALL) [121]. Among them, up to 80% of mutations accumulate within the genomic region encoding its Runt domain, and thereby disrupting its structure [122,123]. Consistent with these observations, *RUNX1*-deficient mice exhibited a significant defect in hematopoiesis [118,119]. Collectively, it is conceivable that RUNX1 acts as a tumor suppressor for myeloid leukemia.

Unlike RUNX1, RUNX2 plays a pivotal role in the regulation of osteoblast differentiation and bone formation [124,125]. Accordingly, RUNX2 transactivates a number of its target genes implicated in osteogenesis such as *type 1 collagen* (*COL1A1*, *COL1A2*), *Osteopontin* (*OCN*, *SPP1*) and *Osteocalcin* (*OPN*, *BGLAP*) [126]. Notably, RUNX2 is also associated with osteosarcoma [127,128]. In support of this notion, amplification of chromosome 6p21, where *RUNX2* exists, was detectable in a subset of osteosarcomas [129]. Several lines of evidence indicate that the dysregulation of *RUNX2* expression is frequently detectable in a variety of human cancers and its higher expression level is tightly correlated with poor clinical outcome of the patients [24,25,26]. Indeed, RUNX2 also transactivates numerous genes involved in carcinogenesis [27,28,29,30,31,32]. Recent studies revealed that RUNX2-mdiated carcinogenesis is dependent on the direct activation of *survivin* expression in prostate cancer cells [33]. *Survivin* is highly expressed in a wide range of human cancers, which correlates with both accelerated relapse and drug resistance [34]. Thus, it is likely that RUNX2 participates in osteogenic differentiation as well as carcinogenesis in a cell context-dependent manner.

It has been well documented that RUNX3 is required for T cell development during thymopoiesis and plays an essential role in the regulation of the dorsal-root ganglion proprioceptive neuron function [130]. In addition to its capability to modulate the lineage-specific gene expression, RUNX3 has been shown to be obviously involved in the formation of a number of cancers including gastric, colorectal, liver, lung and breast cancers [131,132]. For example, Li *et al.* have described that *RUNX3*-deficient mouse gastric mucosa displays hyperplasia due to the enhanced proliferation and suppressed cell death [133]. Based on their results, tumorigenicity of human gastric cancer-derived cells was inversely correlated with *RUNX3* expression level and mutation within the genomic region encoding Runt domain of RUNX3 (R122C) abrogated its tumor suppressive activity. It is worth noting that *RUNX3* expression is kept at an extremely low level both in gastric cancer-derived cells and gastric cancer tissues, which might be due to the hypermethylation of the CpG island of *RUNX3* exon 1 region. Unexpectedly, *RUNX3* is rarely mutated in primary gastric cancer tissues. Therefore, DNA methylation-mediated silencing of *RUNX3* might contribute to the loss of its tumor-suppressive function.

## 6. Functional Collaboration between the p53 Family and RUNX Family during DDR

Considering that the p53 family participates in the regulation of DDR pathway, a clear understanding of the mechanistic basis underlying DNA damage-mediated activation and/or inhibition of p53 family-dependent cell death pathway is of particular interest in overcoming drug-resistant phenotype of malignant cancers [134]. In this connection, we have focused on the functional interaction between p53 family and RUNX family in response to DNA damage. As described previously [135], we have found for the first time that RUNX3 is associated with p53, enhancing p-ATM-mediated its phosphorylation at Ser-15, and thereby stimulating DNA damage-induced cell death. Our subsequent studies demonstrated that RUNX1 acts as a molecular bridge or a scaffolding protein for p53-histone acetyltransferase p300, assists p300-mediated acetylation of p53 at Lys-373/382 and further promotes p53-dependent cell death following DNA damage [136]. These observations strongly suggest that RUNX1 as well as RUNX3 serve as a co-activator for pro-apoptotic p53 during DDR.

**Figure 4:**
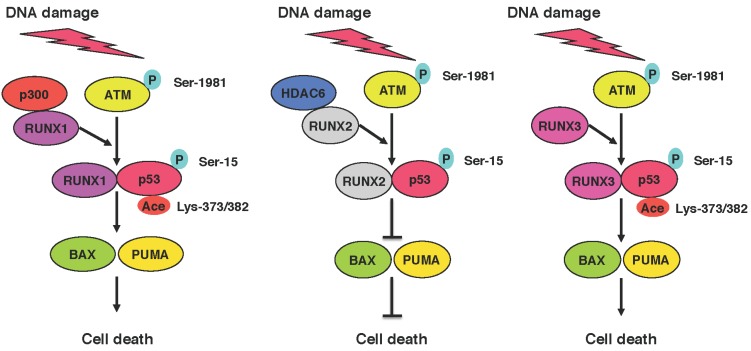
Functional interaction between p53 family and RUNX family. In response to DNA damage, RUNX1 and RUNX3 assist p300-mediated acetylation of p53 at Lys-373/382 and p-ATM-mediated phosphorylation of p53 at Ser-15, respectively, and then augment its pro-apoptotic activity. In contrast, RUNX2 is associated with HDAC6, and thereby prohibits pro-apoptotic activity of p53 through its deacetylation.

In contrast, we have recently found that pro-oncogenic RUNX2 has an inhibitory role in the regulation of DDR [35]. Based on our results, RUNX2 collaborated with histone deacetylase 6 (HDAC6) and then prohibited pro-apoptotic activity of p53 after DNA damage (Figure 4). In addition to p53, we have also revealed that RUNX2 trans-represses DNA damage-mediated up-regulation of *TAp73*, and thereby abrogating TAp73-dependent cell death in response to DNA damage [137]. Moreover, RUNX2 formed a complex with TAp73 and attenuated its transcriptional activity. Thus, RUNX2 might be one of the attractive therapeutic targets for malignant cancer treatment.

## 7. Anti-Cancer Drug Resistance of Cancer Stem Cells

Stem cells (SCs) have the unique property of self-renewal to maintain tissue functions. Their quality must be strictly checked through the specific protective mechanisms that ensure their genomic integrity [138,139]. According to the previous estimation [140], cells including SCs receive 100,000 spontaneous DNA lesions per day. The proper DDR is therefore essential for SCs to prevent dysfunction of specific tissues caused by loss of SC pool and/or carcinogenesis [138]. Recent studies have demonstrated that a small population of cancer cells might possess a highly oncogenic ability when transplanted in immune-deficient mice, and be functionally similar to tissue-specific SCs, so-called cancer stem cells (CSCs). In several malignant cancers including brain, breast and colon cancers, CD133 has been considered to be one of the putative CSCs marker proteins to identify them as well as tissue-specific SCs such as hematopoietic SCs [45,141,142]. In fact, CD133-positive glioma cells are resistant to irradiation [141]. Collectively, increasing evidence indicates that the putative CSC-like cells display resistance to chemotherapy and radiotherapy, implying that CSCs are at least in part responsible for cancer recurrence after treatments [143].

As described [24,25,26], the expression level of *RUNX2* was higher in a variety of human cancer tissues including prostate cancer than that in their corresponding normal ones. Intriguingly, CSCs enriched from prostate cancer tissues highly expressed *RUNX2* as well as its target gene *Survivin* [47,48]. In addition, CD133-positive cells isolated from primary colorectal cancers were able to develop cancers in nude mice [139,142]. Consistently, knockdown of *CD133* suppressed the xenograft tumor formation and growth of spheres in human colon cancer cells [144]. Together, it is likely that RUNX2 as well as CD133 are closely involved in the acquisition and/or maintenance of drug resistance of CSCs.

We and the other groups demonstrated that CD133 triggers the activation of PI3K/AKT pathway and potentiates oncogenic ability of glioma, hepatocellular carcinoma, neuroblastoma and colon cancer cells [145,146,147]. The oncogenic role of PI3K/AKT axis on cancer growth extends beyond its pro-proliferative and survival effects and includes migration as well as invasion. Notably, it has been shown that there exists a functional relationship between pro-oncogenic PI3K/AKT pathway and RUNX2. For example, Su *et al.* found that the constitutively active AKT induces *RUNX2* expression as well as its target genes involved in carcinogenesis [148]. Consistently, Sase *et al.* described that RUNX2 immuno-reactivity in colon cancer cells is associated with their aggressive clinical behavior and its higher expression level is mediated by the stimulation of PI3K/AKT pathway [149]. In support of these observations, the activation of PI3K/AKT pathway augmented the sequence-specific DNA-binding ability of RUNX2 [150], suggesting that RUNX2 serves as a substrate of AKT and also an important mediator of pro-oncogenic PI3K/AKT signaling pathway. Thus, it is plausible that PI3K/AKT/RUNX2 regulatory axis participates in the genesis and/or maintenance of drug-resistant CSCs.

**Figure 5:**
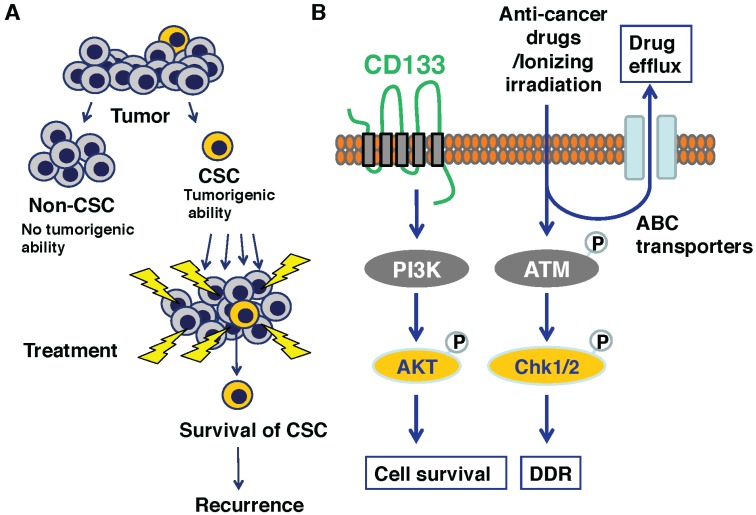
Resistant phenotype of cancer stem cells (CSCs). (**A**) Schematic drawing of CSC hypothesis; (**B**) Major signaling pathways for resistant phenotype of CSCs.

Additionally, it has been shown that a variety of tissue-specific SCs and CSCs enrich in side population (SP) of cells originally identified from hematopoietic SCs with a higher efflux rate of Hoechst 33342 [151,152]. The efflux of dye is largely mediated by ATP-binding cassette (ABC) transporters such as ABCB1 (multidrug resistant 1/MDR1), ABCC1 (MDR protein 1/MRP) and ABCG2 (breast cancer-resistant protein1/BCRP1). These ABC transporters have been well known to pump a number of anti-cancer drugs, and might be implicated in the acquisition and/or maintenance of drug-resistant phenotype of CSCs (Figure 5).

## 8. Attractive Strategies to Overcome Anti-Cancer Drug-Resistant Malignant Cancers

Since MDM2 binds to p53 and abrogates its pro-apoptotic activity, a specific inhibition of the complex formation between MDM2 and p53 might be an attractive strategy to augment pro-apoptotic activity of p53 in response to DNA damage. For example, a small chemical compound termed Nutlin, which binds directly to the p53-binding pocket of MDM2, interrupts p53/MDM2 interaction, and subsequently prohibits MDM2-dependent proteasomal degradation of p53 [153,154]. An alternative approach is mutant p53 reactivation by a small chemical compound, which promotes correct folding of mutant p53. PRIMA-1 and MIRA-1 can restore wild-type conformation to mutant p53, and then induce cell death of cancerous cells [155,156].

**Figure 6:**
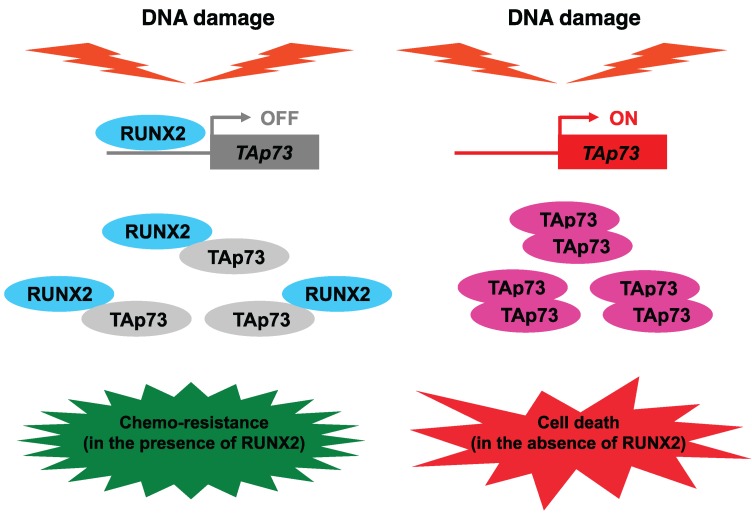
siRNA-mediated silencing of *RUNX2* enhances TAp73-dependent cell death pathway in the absence of functional p53. RUNX2 trans-represses DNA damage-mediated induction of TAp73 and forms a complex with TAp73 to prohibit its transcriptional activity. Depletion of *RUNX2* further enhances TAp73-dependent cell death pathway in response to DNA damage [136].

According to our recent findings, siRNA-mediated knockdown of pro-oncogenic *RUNX2* efficiently augmented p53/TAp73-dependent cell death in *p53*-proficient human osteosarcoma-derived cells following DNA damage [35,136]. Similar results were also observed in *p53*-deficient or *p53*-mutated human pancreatic cancer cells [157], suggesting that siRNA-mediated depletion of *RUNX2* promotes the proper DDR regardless of *p53* status (Figure 6). Unfortunately, it has been well known that siRNA is unstable and its knockdown effect is transient. Recently, Zorde Khvalevsky *et al.* developed a local prolonged siRNA delivery system (termed LODER) [158]. Based on their results, LODER system protected siRNA from degradation and released intact siRNA stably and slowly within cancer cells over a few months. Therefore, this LODER system might overcome the current siRNA delivery obstacles, and siRNA-mediated silencing of *RUNX2* using LODER delivery system is an original and attractive therapeutic strategy to treat drug-resistant malignant cancers.

## 9. Conclusions

An appropriate DDR signaling pathway plays a vital role in the regulation of cell fate determination after DNA damage (cell survival or death). Tumor suppressor p53 has a crucial role in eliminating seriously damaged cells, and thereby maintaining genomic integrity. Therefore, its dysfunction arising from mutations renders cancerous cells resistant to anti-cancer drugs. Indeed, p53 mutant acts as a dominant-negative inhibitor against wild-type p53. In contrast to *p53*, the other *p53* family members such as *TAp73* and *TAp63* are rarely mutated in cancer cells. Importantly, TAp73 and TAp63 are capable of inducing cancer cell death following DNA damage in the absence of functional p53. Consistently, forced expression of TAp73 promotes cell death in *p53*-deficient as well as *p53*-mutated cancerous cells. Meanwhile, RUNX1 and RUNX3 enhance p53-dependent cell death in response to DNA damage. On the other hand, RUNX2 attenuates p53/TAp73-mediated cell death following DNA damage. Thus, silencing of *RUNX2* might be a promising strategy to improve the efficacy of DNA damage-inducing anti-cancer drugs through the activation of the p53 family-dependent cell death pathway.
